# Supplementary material for: Long-read sequencing identifies aberrant fragmentation patterns linked to elevated cell-free DNA levels in cancer
Source: Genome Biol. 2026 Apr 10;27:165. doi: 10.1186/s13059-026-04060-8 (PMC13173744; doi:10.1186/s13059-026-04060-8)
Supplement: Supplementary file 1 — Additional file 1: Seven Supplementary Figures S1-S7. [file 13059_2026_4060_MOESM1_ESM.docx]

**Supplemental Figures for:
Long-read sequencing identifies aberrant fragmentation patterns linked to elevated cell-free DNA levels in cancer**

Benjamin P. Berman^1,2,*^, Sarah A. Erdman^1^, Christina Wheeler^1^, Justin Cayford^1^, Jean-Valery Turatsinze^3,4^, Maria Ouzounova^5^, Marie Piecyk^6,7^, Marielle Herzog^3^, Léa Payen-Gay^6-8^, Thomas Walter^5,9^, Theresa K. Kelly^1,*^

1. Volition America LLC, Henderson, Nevada, United States of America

2. Department of Developmental Biology and Cancer Research, The Hebrew University of Jerusalem, The Institute for Medical Research Israel-Canada, Jerusalem, Israel

3. Belgian Volition SRL, Parc Scientifique Crealys, Isnes, Belgium

4. Current address: Diagenode/Hologic, Belgium

5. Gastroenterology and technologies for health (Université Claude Bernard Lyon 1, INSERM U1052, CNRS UMR5286, Centre Léon Bérard), Cancer Research Center of Lyon, Lyon, France

6. Center for Innovation in Cancerology of Lyon (CICLY) EA 3738, Faculty of Medicine and Maieutic Lyon Sud, and Institute of Pharmaceutical and Biological Sciences (ISPB), Claude Bernard University Lyon I, 69921 Oullins, France.

7. Department of Biochemistry and Molecular Biology, Lyon-Sud Hospital, Hospices Civils de Lyon, 69495 Pierre-Bénite, France.

8. Toxicology department, Institute of Pharmacy and Biology of Lyon (ISPB), Claude Bernard University Lyon I 8 avenue Rockefeller 69008 Lyon.

9. Department of Medical Oncology, Edouard Herriot Hospital, Hospices Civils de Lyon, Lyon, France

* Co-supervisors of the study
Correspondence to: b.berman@volition.com, t.kelly@volition.com


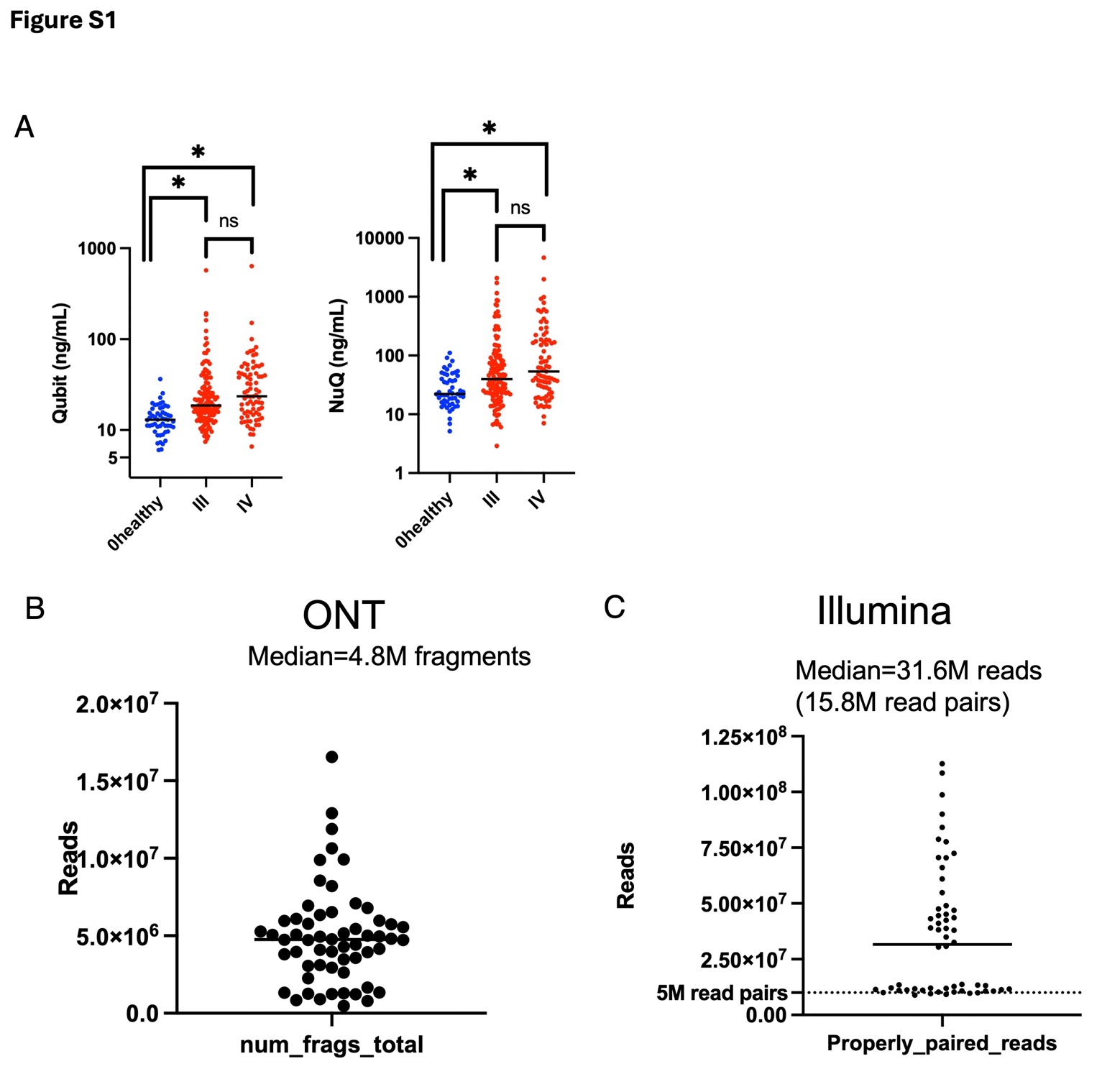


**Figure S1:** (A) cfDNA levels (left) and H3.1 nucleosome levels (right) in the screening dataset of 236 cancer samples and 52 healthy samples. (B) Read counts for 58 cancer and healthy samples from the pan-cancer healthy cohort, sequenced using ONT MinION Mk1c. Each fragment consists of a single read. (C) Read counts for the matched 58 cancer and healthy samples sequenced using Illumina NovaSeq. Each fragment consists of a single read pair. Panels A-B were performed using a two-tailed, unpaired t-test. Panel A p-values 0.015 (healthy vs. Stage III) and 0.011 (healthy vs. Stage IV), panel B p-values 0.011 (healthy vs. Stage III) and 0.016 (healthy vs. Stage IV).


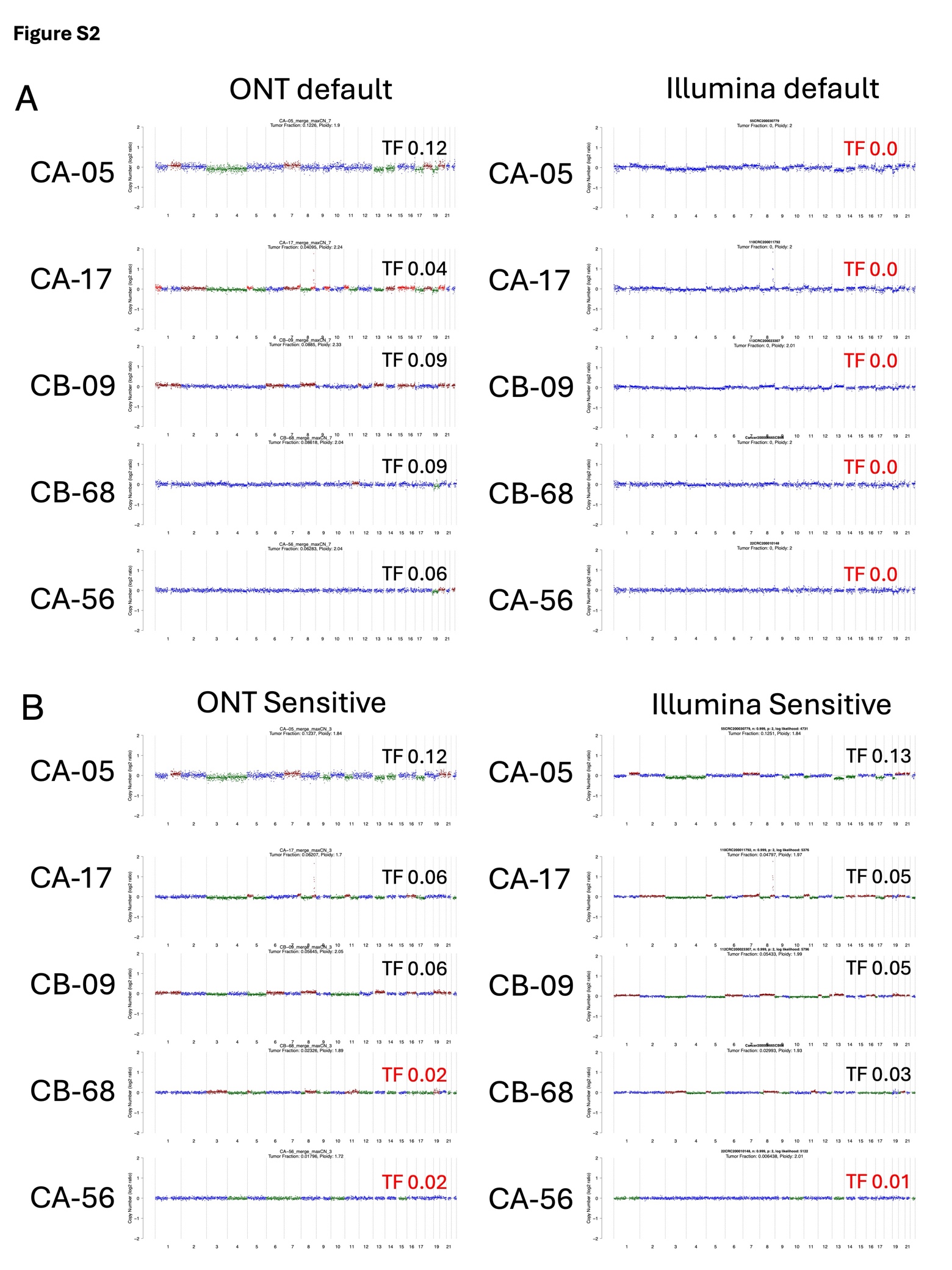


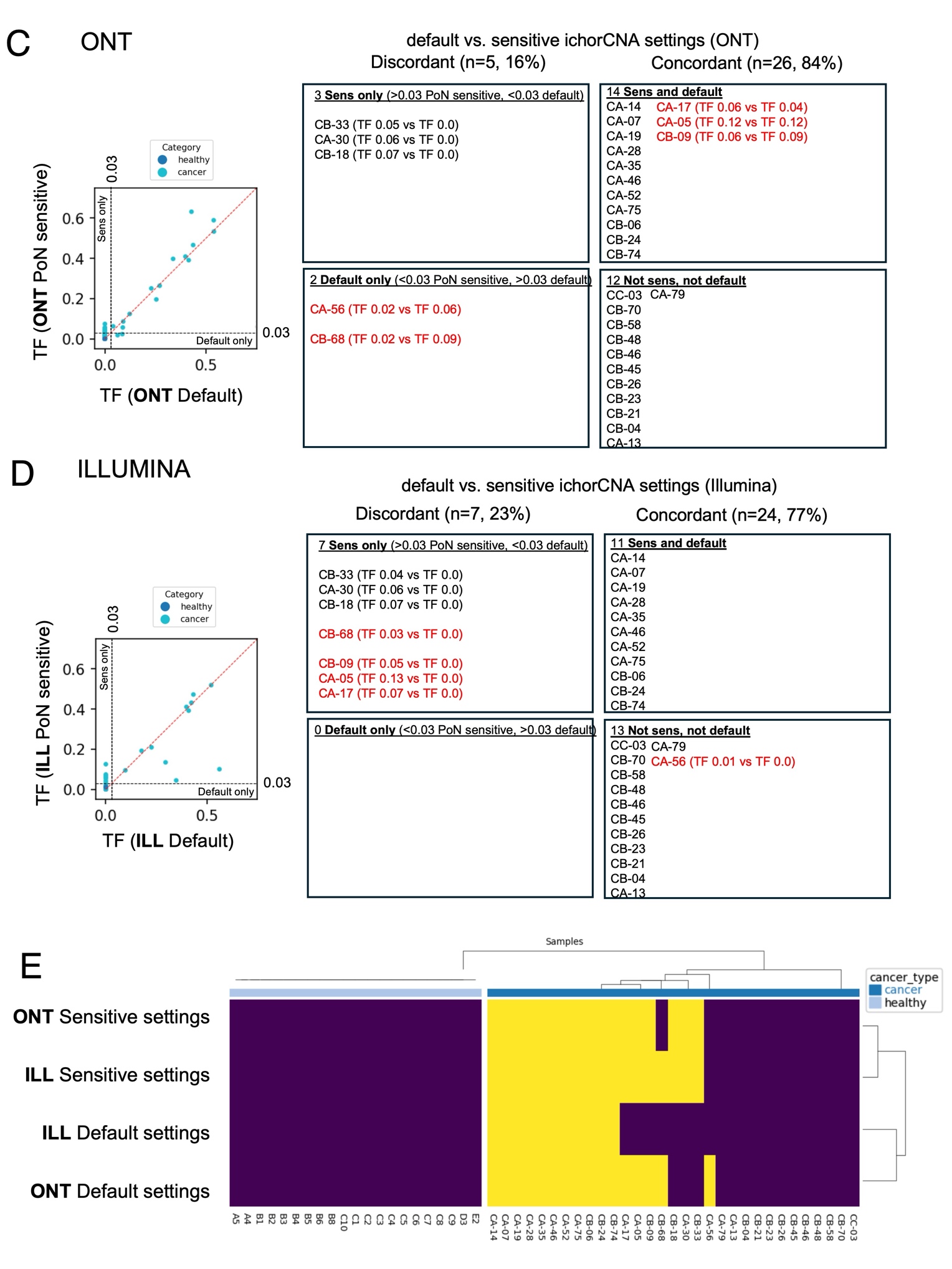


**Figure S2: ichorCNA default settings vs. sensitive settings.** (A) ichorCNA copy number plots for five samples that were only called by ONT and not by Illumina using ichorCNA default settings (“ONT-only” cases). (B) Same five samples, with ichorCNA sensitive settings. “TF” refers to estimated tumor fraction. (C) Differences in ONT samples between default and sensitive settings, for the 31 cancer samples run on both platforms. Scatter plot on left shows estimated tumor fractions, with dotted lines drawn at 0.03, the recommended limit of detection for ichorCNA. Boxes on the right are based on binarizing each sample as detected vs. not detected in each condition and dividing samples into boxes based on those samples that are discordant between default and sensitive settings (5 samples) and those that are concordant (26 samples). Cases listed in red are those that change boxes in the Illumina data below. (D) Same as C, but based on the Illumina samples. Red samples are those that are assigned to different boxes in the ONT analysis in C. (E) is a summary of C and D, where blue boxes have tumor fraction below the level of detection, and yellow boxes are above the level of detection.


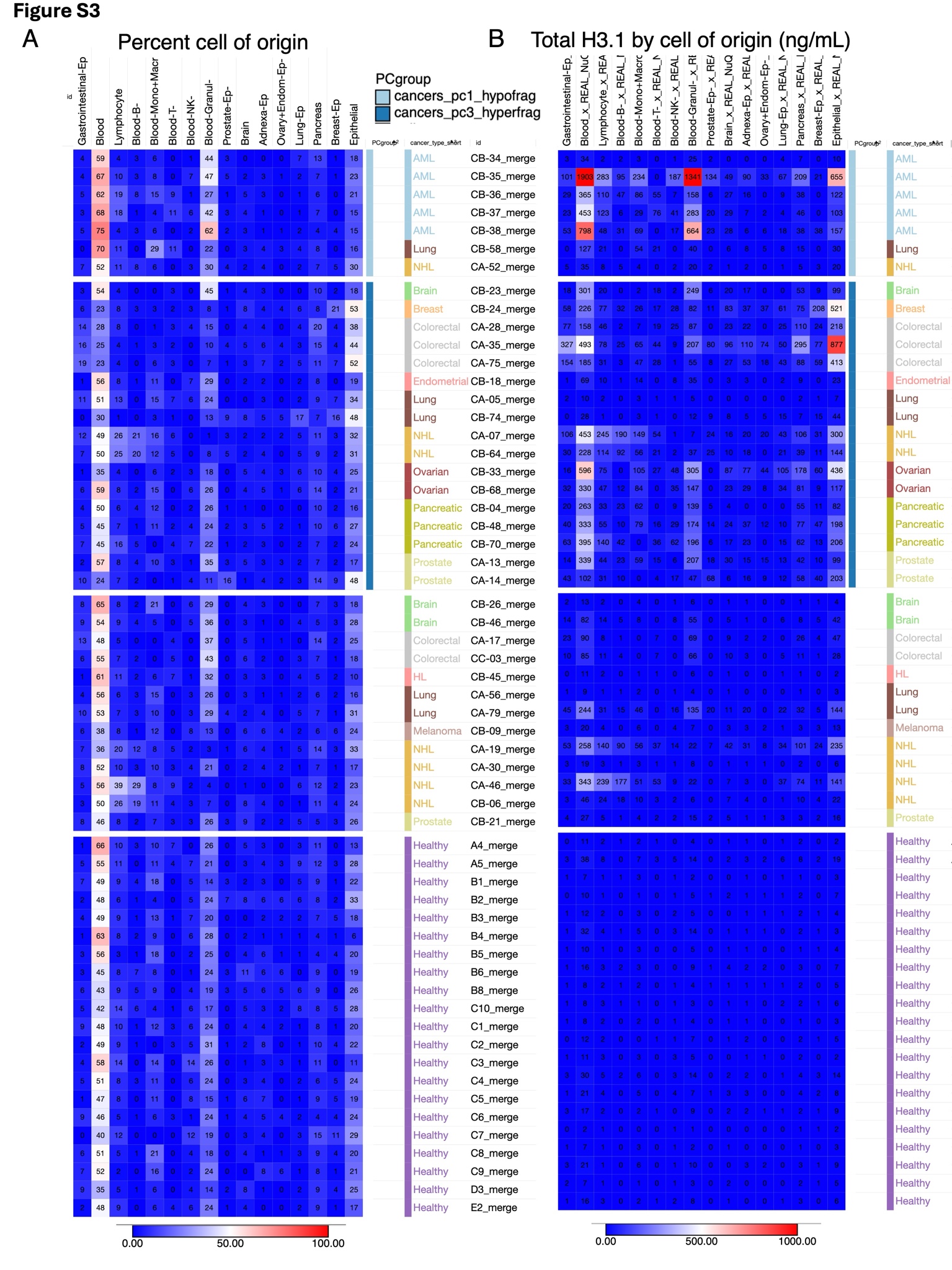


**Figure S3: Methylation-based cell of origin (COO)**. (A) For each sample, the non-negative least squares (NNLS) method of CelFiE-ISH was used to estimate the percentage of the mixture derived from each of 31 individual cell types. We then combine several cell type groups by summing percentages, with cell type groups as follows:

Gastrointestinal-Ep: 'Gastric-Ep','Colon-Ep','Small-Int-Ep'
Blood: 'Blood-T','Blood-NK','Blood-Mono+Macro','Blood-Granul','Blood-B','Eryth-prog'
Lymphocyte: 'Blood-T','Blood-NK','Blood-B'
Adnexa-Ep: 'Ovary+Endom-Ep','Fallopian-Ep'
Lung-Ep: 'Lung-Ep-Bron','Lung-Ep-Alveo'
Pancreas: 'Pancreas-Duct','Pancreas-Acinar','Pancreas-Delta','Pancreas-Beta','Pancreas-Alpha'
Breast-Ep: 'Breast-Basal-Ep','Breast-Luminal-Ep'
Epithelial: 'Bladder-Ep', 'Breast-Basal-Ep', 'Breast-Luminal-Ep', 'Colon-Ep', 'Fallopian-Ep', 'Gastric-Ep', 'Head-Neck-Ep', 'Kidney-Ep', 'Lung-Ep-Alveo', 'Lung-Ep-Bron', 'Ovary+Endom-Ep', 'Prostate-Ep', 'Small-Int-Ep', 'Thyroid-Ep'

Since these groups are not mutually exclusive, the percentages do not sum to 100%, although the 31 primary cell type percentages do. For instance, Blood-B is shown as an individual cell type but also contained in both “Lymphocyte” and “Blood”. (B) Each cell type or cell type group percentage is multiplied by the total concentration of H3.1 nucleosomes (ng/mL) for the sample. The color scale is trimmed at a maximum value of 1,000 ng/mL.

**
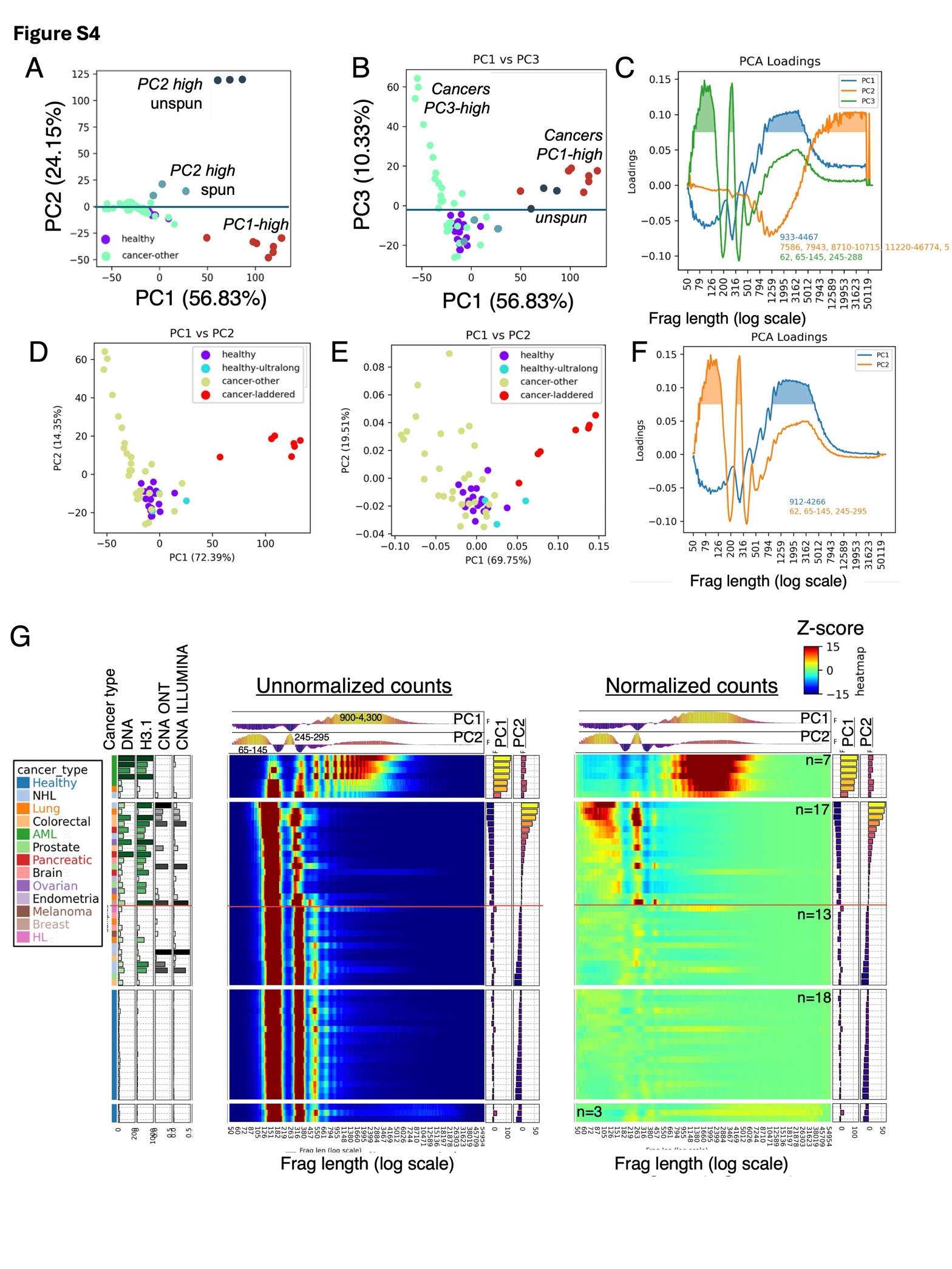
**

**Figure S4: Principal Component Analysis of fragment length profiles.** (A-C) correspond to the PCA presented in the main Figure 4. For this analysis, 58 healthy and cancer samples were included, plus the “unspun” samples of the 3 healthy volunteers with ultra-long fragments (E2, C10, D3). The input vector for each sample included one value for each fragment length bin, which represented the fraction of DNA in that bin, z-score normalized by the mean and standard deviation of the fractions of all healthy samples for the same bin (not including the “unspun” samples). (A-B) PC1, PC2, and PC3 values for each sample. In (B) a line is drawn between the PC3-high hyperfragmented cancer group and the normal-like cancer group in the main Figure 4. (C) shows the PCA loadings for each bin for each of the top 3 PCs. An arbitrary cutoff of 0.075 was used to define the bin ranges strongly associated with each PC, which are listed inside the figure. (D-G) An alternative PCA where the 3 unspun samples were omitted. The PC1 vs. PC2 plot in (D) is nearly identical to the PC1 vs. PC3 plot in the (B) panel. (E) An alternative PCA where the 3 unspun samples were omitted, and raw percent of DNA values were used rather than z-score normalized versions. (F) PCA loadings, defining the bin ranges strongly associated with each of the top 2 PCs, using the same cutoff as the (C) panel. (G) Sample ordering based on the alternative PCA.

**
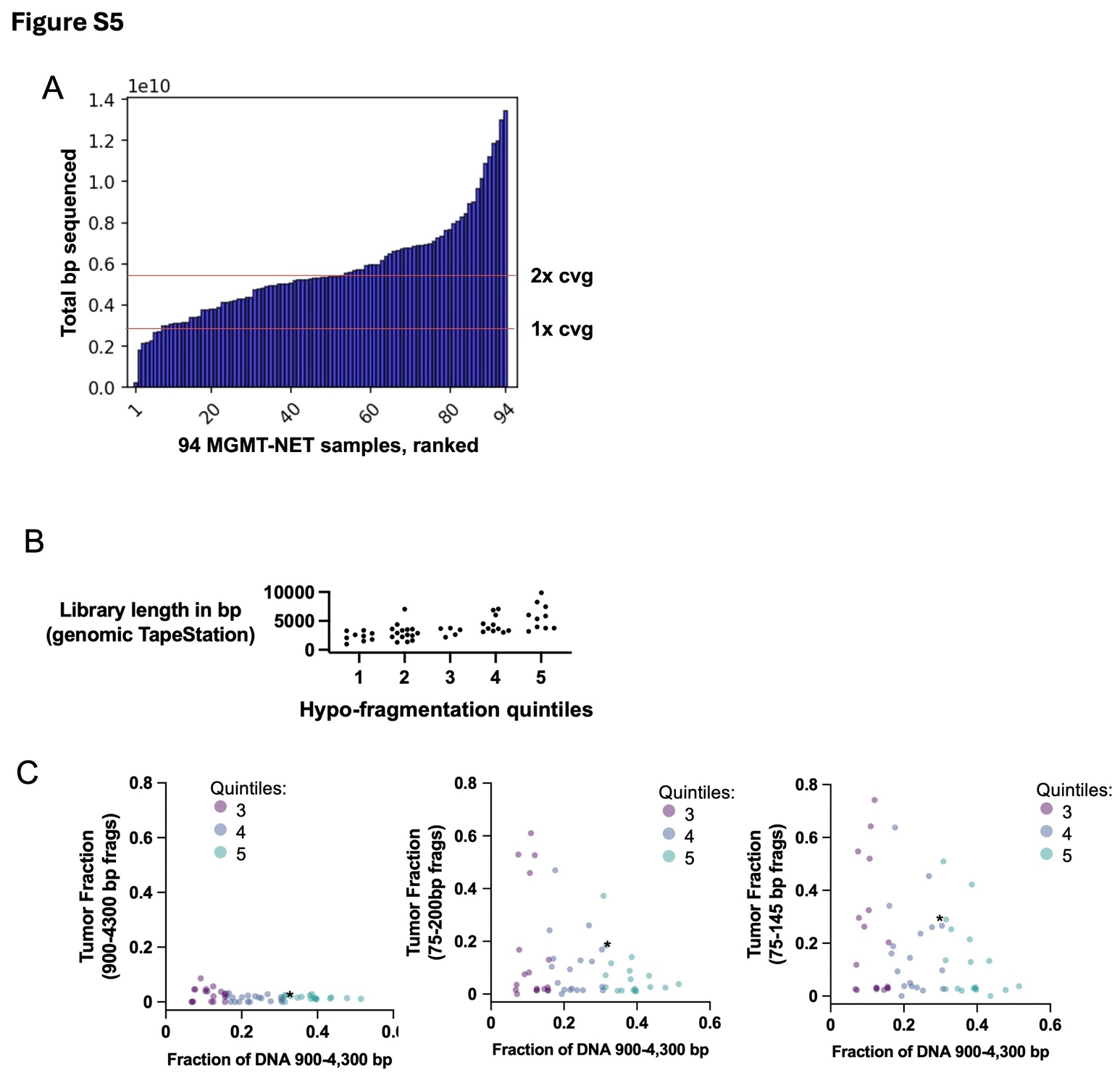
**

**Figure S5: ONT neuroendocrine cohort.** (A) 94 sequenced samples, ordered by the total number of base pairs in all sequenced reads. 5 samples were not included in subsequent analysis. (B) Samples where pre-sequence DNA sizing was performed using the “genomic” tape for the Agilent TapeStation system. Quintiles are those defined in Figure 5, from the least hypofragmented (quintile 1) to the most (quintile 5). (C) Hypofragmentation (Fraction of DNA in fragments 900-4,300bp) is plotted against ichorCNA tumor fraction estimates. In each case, the ichorCNA analysis is performed with a specific set of reads (900-4300bp fragments on the left, 75-200bp fragments center, and 75-145 bp fragments right). Coloring is based on the same hypo-fragmentation quintiles from Figure 5.

**
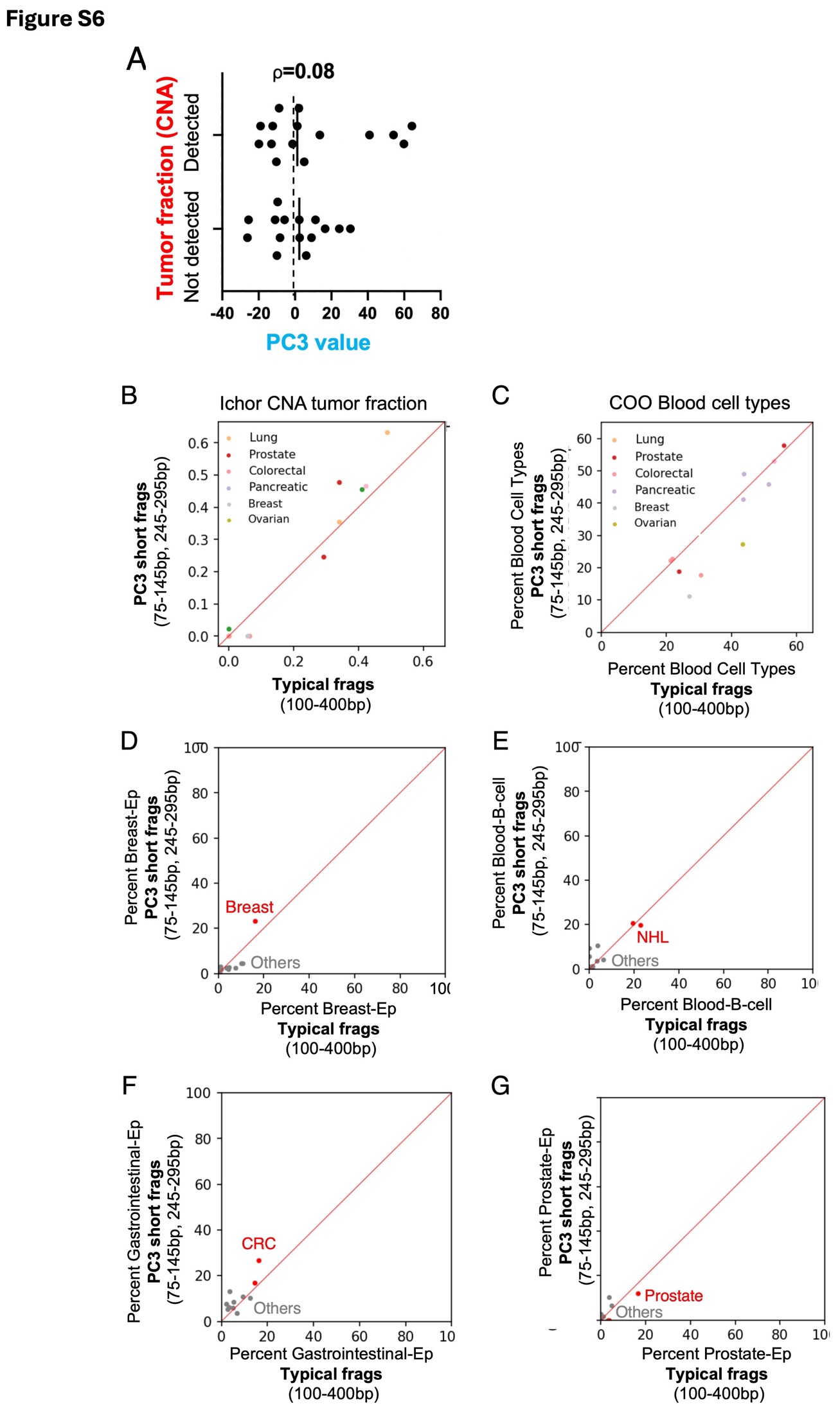
**

**Figure S6: Associations between hyperfragmentation and cancer origin.** (A) Alternative binarized version of the binarized data shown in Figure 5E. Each point is one of 30 cancer samples (the 7 PC1-high hypofragmented cancer samples are omitted). The y axis is the tumor fraction estimated from ichorCNA based on the ONT sequence data. (B-G) Cancer cell fraction in shortened fragments in hyperfragmented samples. Of the 17 PC3-high samples (PC3>-2), 7 had less than 1 million fragments in either of the PC3-associated length ranges 75-145 or 245-295. We used the remaining 10 to perform ichorCNA and methylation-based cell of origin (COO) analysis. (A) ichorCNA was run using only typical fragments (100-400bp) and fragments in the PC3-associated length ranges. (B-F) Methylation cell of origin was computed for combined Blood cell types (B) as well as cell types which represented the correct cell of origin: Breast-Ep for Breast cancer (C), B-cell for Non-Hodgkins Lymphoma (D), Gastrointestinal-Ep for Colorectal cancer (E), and Prostate-Ep for Prostate Cancer (F)

**
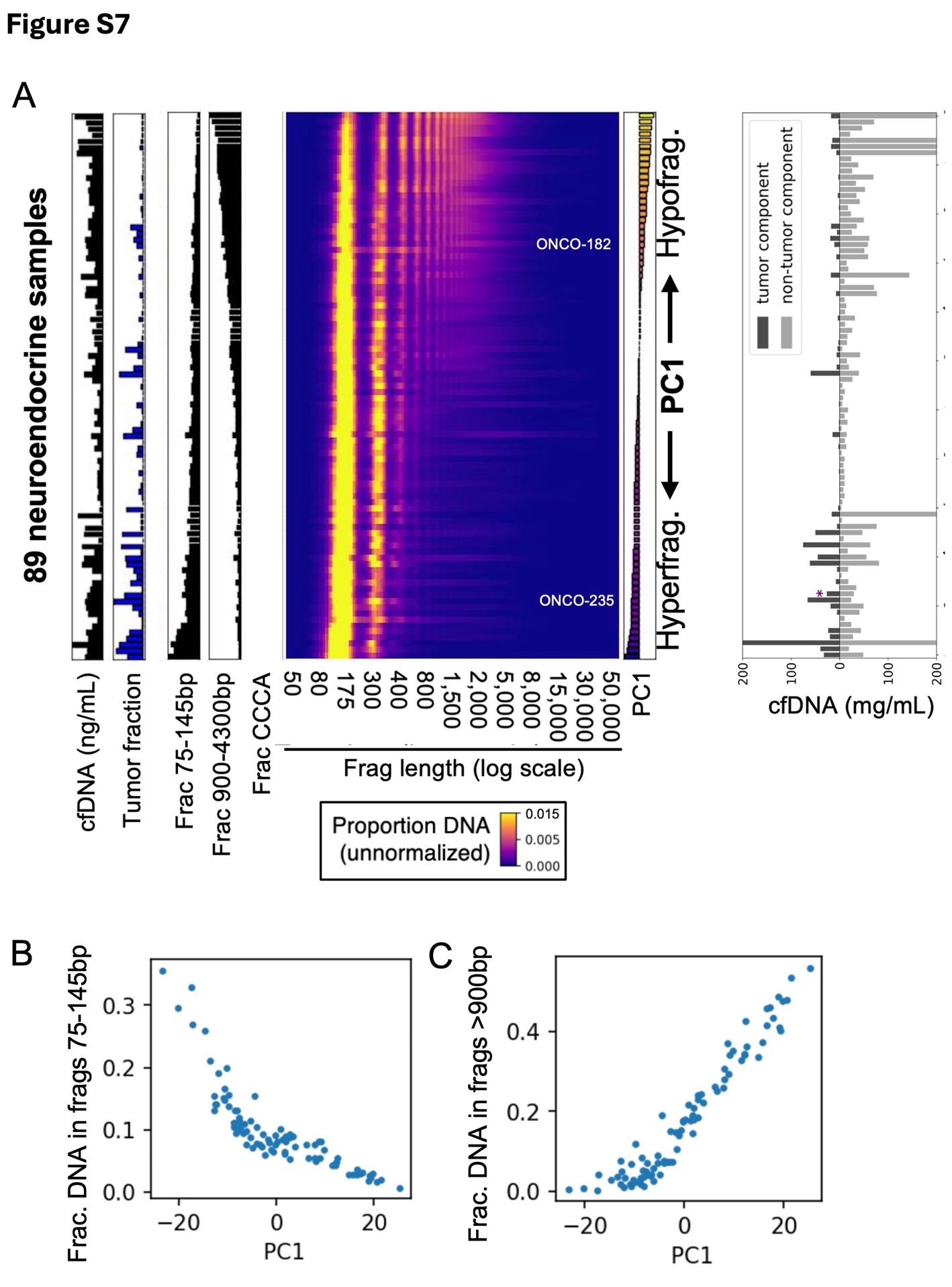
**

**Figure S7: Principal Component Analysis of neuroendocrine samples based on fragment length**. Samples are ordered by the first component (PC1), which separates hyperfragmented samples (bottom) from hypofragmented samples (top). (B-C) PC1 value plotted against the fraction of DNA in short fragments (B) vs. long fragments (C).
